# Eosinophilic fasciitis*

**DOI:** 10.1590/abd1806-4841.20164683

**Published:** 2016

**Authors:** Elisa Baranski Lamback, Fernanda Simões Seabra Resende, Thiara Cristina Rocha Lenzi

**Affiliations:** 1 Hospital Federal da Lagoa – Rio de Janeiro (RJ), Brazil; 2 Hospital Regional da Asa Norte (HRAN) – Brasília (DF), Brazil; 3 Escola Superior de Ciências da Saúde (ESCS) – Brasília (DF), Brazil

**Keywords:** eosinophilia, fasciitis, fibrosis

## Abstract

Eosinophilic fasciitis is a rare sclerodermiform syndrome of unknown etiology. It is characterized by the thickening of the muscular fascia and subcutaneous tissue, with a variable infiltration of eosinophils. Peripheral eosinophilia, poly or monoclonal hypergammaglobulinemia and increased erythrocyte sedimentation rate can be seen. Clinical features begin acutely, with local edema and a painful and symmetrical stiffening of the limbs, progressing rapidly to fibrosis, which can limit joint movements. Some cases have a history of strenuous physical exercise or trauma. The diagnosis is confirmed by a deep skin biopsy. Glucocorticoids in high doses is the treatment of choice. We report a typical eosinophilic fasciitis case with peripheral eosinophilia and dramatic response to pulse therapy with methylprednisolone.

## INTRODUCTION

Eosinophilic fasciitis (EF), or Shulman syndrome, was first described in 1974 as a variant of scleroderma with eosinophilia and fasciitis. However, the absence of sclerodactily, Raynaud’s phenomenon, visceral involvement and a good response to systemic therapy with glucocorticoids distinguished this syndrome as its own entity. It is a rare disease of unknown etiology, with less than 300 cases reported.

## CASE REPORT

**Figure 1:**
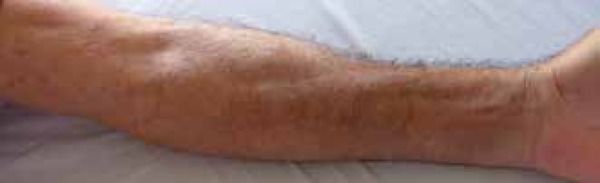
Linear depression that follows the path of the vessels in the upper left limb

**Figure 2:**
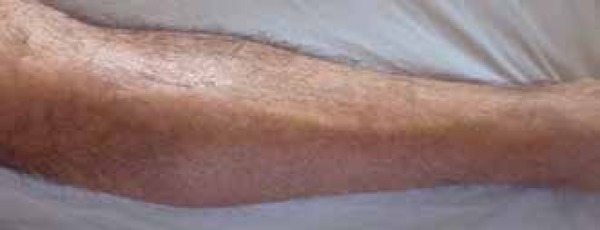
Skin stiffness in the lower right limb Stiff, firmly adherent skin

A 44-year-old, previously healthy, male patient, presented with painful and symmetrical edema in lower limbs, with subsequent local stiffening of the skin. In eight months, the symptoms progressed to the upper limbs and the lower abdomen. He denied any recent strenuous physical effort or trauma. Physical examination showed a depression along the course of superficial veins (*Groove sign*) in the upper limbs and stiffening of the skin in the lower limbs and lower abdomen (Figures 1 and 2). His face and fingers were not affected.

**Figure 3:**
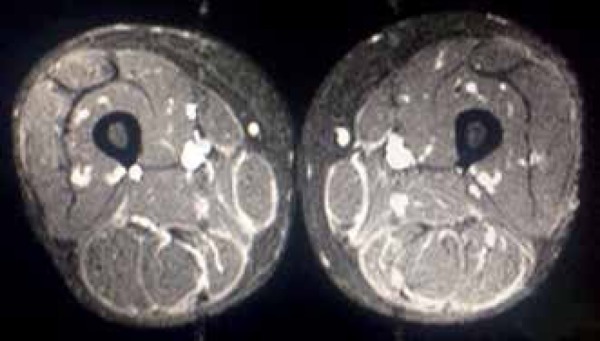
MRI of the lower limbs, axial view Thickening of fascia in medial and posterior areas of the lower limbs

Laboratory tests revealed peripheral eosinophilia of 1600/uL, monoclonal IgG hypergammaglobulinemia, and negative antinuclear antibodies (ANA) and anti-SCL 70. Blood count, hepatography, thyroid function, creatine kinase and lactate dehydrogenase were normal. Magnetic resonance imaging (MRI) showed thickening and enhancement of the fascia in the medial and posterior muscle compartments of the lower limbs (Figure 3).

**Figure 4:**
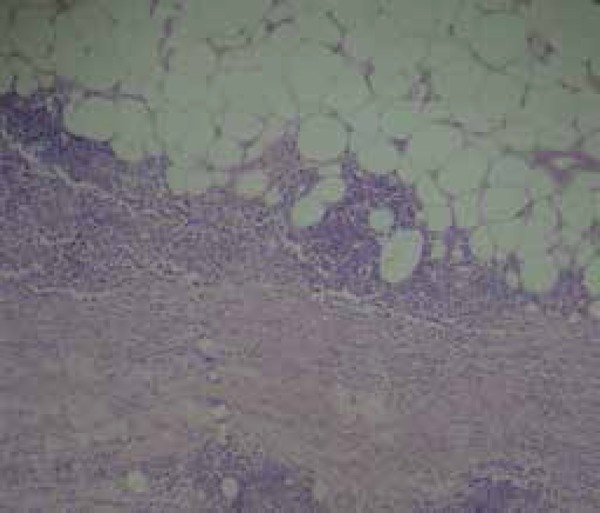
Histopathology - Chronic Fasciitis Thickened and hyalinized fascia, permeated by mononuclear cell infiltration (HE staining, 100X)

A biopsy of the skin and the right forearm muscle showed thickened, hyalinized fascia, permeated by moderate mononuclear inflammatory infiltrate (lymphocytes, plasma cells and histiocytes) and interstitial edema, confirming the diagnosis of chronic fasciitis (Figure 4).

The patient was hospitalized for pulse therapy with methylprednisolone 1g/day for three consecutive days and showed significant improvement in skin thickening and joint mobility in the first week after the pulse therapy. The patient was discharged with prednisone 60 mg/day and methotrexate 15mg/week, with gradual dose reduction and eventual suspension of medications after one year of treatment. Two years after his hospitalization, no signs or symptoms of the disease have been shown, and no medication has been prescribed.

## DISCUSSION

EF is a rare disease that primarily affects adults, between 40 and 50 years of age, with no gender predilection, but presumably affecting men earlier, with less than 300 reported cases to date.^1,2^ Its etiology remains unknown, and a recent history of strenuous exercise or trauma is seen in 30 to 46% of cases.^3-4^

Classically, EF begins with edema, erythema and subsequent painful and symmetrical tightening of the limbs, which ressembles an orange peel texture. The disease progresses for weeks to months, showing fibrosis, hyperpigmentation and woody appearance of the skin, which leads to flexion contractures and decreased mobility. EF typically presents with a linear depression that follows the path of the vessels in the affected area, know as *Groove sign*.^5-6^ The presence of a *Groove sign* and the absence of sclerodactily or Raynaud’s phenomenon distinguishes EF from scleroderma. The face and hands are commonly unaffected.^4,6^

Laboratory findings include peripheral eosinophilia in 60-90% of cases^1^, hypergammaglobulinemia and increased erythrocyte sedimentation rate (ESR).^7^ No antibodies are detected, such as ANA or anti-SCL 70. Eosinophil counts may be very high, but are not associated with disease prognosis.^8^

Skin biopsy, which must be deep and include the muscle, confirms the diagnosis. The fascia is thickened, two to 15 times the normal size, well-defined and attached to the epimysium, with focal or diffuse perivascular inflammation of lymphocytes in most cases, and eosinophils in 69 to 75% of cases, with no necrotic vascular injury.^3^ The eosinophils are not always present in the histopathology, and their absence does not exclude the diagnosis.^1^ Subsequently, the inflammatory changes are replaced by generalized sclerosis, thickening of the fascia and of the underlying tissue layers, with the presence of collagen bands parallel to the fascia and small stripes of adipose tissue between these bands. The epidermis is preserved or rarely atrophic.^2^

MRI is also useful in the diagnosis, showing enhanced fascia, as well as in monitoring the disease response to treatment.^9,10^

Spontaneous resolution occurs in 10-20% of patients after two to five years of disease; in the other cases, treatment should include physical therapy associated with immunomodulatory medication.^7^ High doses of glucocorticoid (equivalent dose of 1 mg/kg/day of prednisone) are described as first-line treatment. In recurrent cases or incomplete response, hydroxychloroquine can be associated with cyclosporine A. The combination of methotrexate with systemic glucocorticoids may be recommended, with full resolution of the disease seen within 12-36 months. ^6,9^

The authors describe a classic case of EF with a favourable outcome and dramatic response to pulse therapy with methylprednisolone. Encountered with a patient who presents with hard and painful edema in the limbs, peripheral eosinophilia, increased ESR and hypergammaglobulinemia, it is important to consider EF in the differential diagnosis. Early diagnosis and prompt treatment of EF may have a positive impact on the patient’s morbidity, quality of life, and even on the disease remission.
